# Retraction

**DOI:** 10.1002/fsn3.2635

**Published:** 2021-11-01

Retraction: Zhang X, Zhang J, Chen T. An ANP‐fuzzy evaluation model of food quality safety supervision based on China's data. Food Sci Nutr. 2020;8: 3157–3163. https://doi.org/10.1002/fsn3.1561. The above article from Food Science & Nutrition, published online on 05 June 2020 in Wiley Online Library (http://onlinelibrary.wiley.com/), has been retracted by agreement between the authors, the Editor‐in‐Chief, Martin Lo, and Wiley Periodicals LLC. The retraction has been agreed due to an error in Formula 2 which invalidates the results of the study.

